# Engineering *Yarrowia lipolytica* to enhance lipid production from lignocellulosic materials

**DOI:** 10.1186/s13068-018-1010-6

**Published:** 2018-01-22

**Authors:** Xochitl Niehus, Anne-Marie Crutz-Le Coq, Georgina Sandoval, Jean-Marc Nicaud, Rodrigo Ledesma-Amaro

**Affiliations:** 1 Micalis Institute, INRA, AgroParisTech, Université Paris-Saclay, 78350 Jouy-en-Josas, France; 2 Industrial Biotechnology, Centro de Investigación y Asistencia en Tecnología y Diseño del Estado de Jalisco (CIATEJ) A.C., 44270 Guadalajara, Jalisco Mexico; 3 Department of Bioengineering, Imperial College London, London, SW7 2AZ UK

**Keywords:** *Yarrowia lipolytica*, Xylose utilization, Acetyl-CoA, Microbial lipids, Metabolic engineering, Synthetic biology

## Abstract

**Background:**

*Yarrowia lipolytica* is a common biotechnological chassis for the production of lipids, which are the preferred feedstock for the production of fuels and chemicals. To reduce the cost of microbial lipid production, inexpensive carbon sources must be used, such as lignocellulosic hydrolysates. Unfortunately, lignocellulosic materials often contain toxic compounds and a large amount of xylose, which cannot be used by *Y. lipolytica*.

**Results:**

In this work, we engineered this yeast to efficiently use xylose as a carbon source for the production of lipids by overexpressing native genes. We further increased the lipid content by overexpressing heterologous genes to facilitate the conversion of xylose-derived metabolites into lipid precursors. Finally, we showed that these engineered strains were able to grow and produce lipids in a very high yield (lipid content = 67%, titer = 16.5 g/L, yield = 3.44 g/g sugars, productivity 1.85 g/L/h) on a xylose-rich agave bagasse hydrolysate in spite of toxic compounds.

**Conclusions:**

This work demonstrates the potential of metabolic engineering to reduce the costs of lipid production from inexpensive substrates as source of fuels and chemicals.

**Electronic supplementary material:**

The online version of this article (10.1186/s13068-018-1010-6) contains supplementary material, which is available to authorized users.

## Background

Microbial lipids have gained much attention during the last few years due to their potential to replace petroleum as a main source of fuels and chemicals. Lipids and lipid-derived molecules can be used in industry to produce biodiesel, biokerosene, pharmaceuticals, nutraceuticals, cosmetics, lubricants, or plasticizers.

Among the most studied microorganisms that are able to produce large amounts of lipids, we find *Yarrowia lipolytica*, a dimorphic yeast with a known genome and convenient molecular tools for its manipulation [1]. In recent years, the lipid metabolism of this yeast has been rationally modified by metabolic engineering in order to maximize lipid production, mainly using glucose as carbon source. Overexpression of the genes directly involved in fatty acid or TAG synthesis [2, 3] and in the supply of NADPH (required cofactor for fatty acid biosynthesis) [4] or deletion of the genes involved in competing pathways, such as beta-oxidation or TAG remobilization [5], has generated strains that can accumulate up to 90% of their DCW as fatty acids [6] or that can reach a yield of 84.7% of the theoretical maximal yield [7]. However, the bioconversion of glucose into lipids by *Y. lipolytica* is still a rather expensive process, which limits its industrial viability. Therefore, novel synthetic biology approaches have been recently developed in order to achieve profitable production of biolipids. Such approaches include the following: (1) the engineering of this yeast to enable fatty acid secretion into the culture media, which facilitates downstream processes (extraction and separation of the lipids); these downstream processes can be up to 60% of the total cost of the process [8]; (2) the modification of this yeast in order to use unusual nitrogen or phosphorus sources, which makes it competitively grow in non-sterilized media, limiting the risk of contamination and therefore reducing the cost associated with sterilization [9]; (3) the heterologous expression of enzymes that enable *Y. lipolytica* to produce specific, more expensive final products (either high-value fatty acids or fatty acid-derived compounds), such as hydroxylated fatty acids [10] or fatty alcohols [11]; and, finally, (4) the expansion of the range of substrates that this yeast can use as carbon source, allowing it to use waste products or low-cost substrates, such as raw starch [12], xylose [13], fructose [14], galactose [15], or other convenient substrates [16].

In this regard, one of the most preferred substrates for biotechnology is lignocellulosic hydrolysate, which is a raw mixture of nutrients that is normally enriched in C6 and C5 sugars, mainly glucose and xylose, which originate from the degradation of cellulose and hemicellulose from plant biomass. The hydrolysates often have a significant amount of potentially toxic compounds, such as organic acids and furfurals [17], which are usually detrimental to most organisms. *Y. lipolytica*, often found in contaminated soils, has the potential to resist such harsh conditions. Moreover, *Y. lipolytica* is able to use glucose very efficiently for the production of lipids. However, the wild-type strains of *Y. lipolytica* cannot use xylose, which can be up to 50% of the components in lignocellulosic material [18].

Therefore, in the last year, several metabolic engineering approaches have been carried out in order to allow *Y. lipolytica* to use xylose. Interestingly, it seems that different genetic backgrounds lead to variability in the capacity of the strains to use this sugar after engineering. Po1d was able to use xylose efficiently after the expression of the heterologous *XDH* (xylitol dehydrogenase) and *XR* (xylose reductase) from *S. stipitis* and the overexpression of the native *XK* (xylulokinase) [13]. The modified strains were optimized to maximize two biotechnological products, lipids and citric acid. However, Po1g expressing the three heterologous genes (*XR*, *XDH*, and *XK*) was unable to grow on xylose until a starvation adaptation was performed [19]. In the case of Po1f background, the overexpression of native genes XK and XDH was enough to permit growth in xylose, although its growth was reduced compared to growth on glucose and especially under nitrogen limitation, which is a required condition to overproduce fatty acids [20]. In previous studies, unmodified Po1g, which is unable to use xylose alone, was shown to produce lipids in detoxified hydrolysates supplemented with peptone (rice bran [21] and sugarcane bagasse [22] hydrolysates). However, no strain modified to fully assimilate xylose has yet been tested for lipid production in complex, non-detoxified, and inexpensive lignocellulosic hydrolysates.

In this work, we re-engineer Po1d and a lipid overproducer derivative strain to efficiently use xylose, even under nitrogen limitation conditions, using native genes. The modified strains were able to produce high amount of lipids from C5. These strains were further engineered in order to convert xylose into acetyl-CoA using alternative pathways by expressing heterologous genes. These modifications increased lipid production. Finally, the engineered *Y. lipolytica* strains were used to produce lipids from a low-cost lignocellulosic hydrolysate in a fed-batch fermentation, which reached to the highest lipids yields described so far.

## Results and discussion

### The overexpression of native genes ylXDH, ylXR, and ylXK is sufficient for efficient growth in xylose

**Fig. 1:**
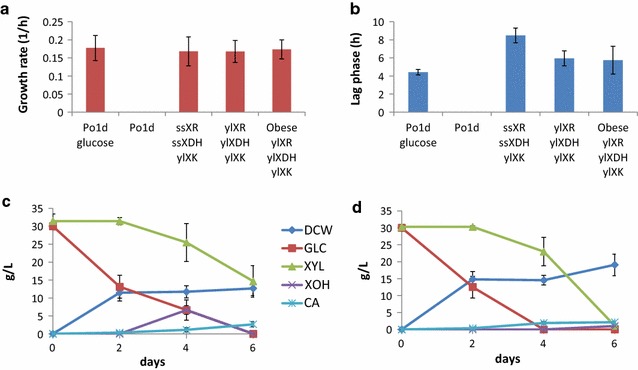
Growth of different strains in xylose or a mixture of glucose and xylose. Bar plot representation of the growth rate (**a**) and the lag phase (**b**) of different *Y. lipolytica* strains. The absence of a bar means that no growth was detected. The values represent the average growth rate (1/h) or lag phase (h) and the standard deviation of at least two replicates growing in microplates. The strain named Po1d is a prototroph-derived strain. Minimal media was used with xylose as a carbon source, unless the carbon source is specified in the figure as glucose. Behavior of the strains ylXYL+ (**c**) and ylXYL+Obese (**d**) growing in a mixture of glucose and xylose. The graphics show the consumption of substrates (xylose and glucose, g/L), DCW (g/L), and production of metabolites (xylitol-XOH and citric acid-CA, g/L)

Recently, we found that *Y. lipolytica* Po1d was able to growth in xylose due to the overexpression of *XDH* and *XR* from *S. stipitis* and native *XK*. Here, we investigate the ability of native *XDH* and *XR* to allow growth in xylose. With this aim, we amplified these two genes and placed them under the control of the strong, constitutive *TEF* promoter. The modified strain, which contains the overexpression cassettes of yl*XDH*, yl*XR*, and yl*XK*, was able to grow in xylose as well as the wild type grows in glucose (similar growth rate), and it grew even slightly better than the strain overexpressing the heterologous genes (shorter lag phase) (Fig. 1a, b; Additional file 1: Figure S1). This strain will be referred to in the paper as ylXYL+.

In parallel, we overexpressed the three native genes in a Po1d-derived background that was previously modified to maximize lipid production through overexpression of genes *DGA2* and *GPD1* from the lipid biosynthetic pathway and deletion of the *POX1*-*6* genes and the *TGL4* gene, which are involved in the degradation and remobilization of lipids in *Y. lipolytica* [14]. The modified strain, named ylXYL+Obese, was able to grow in xylose as a sole carbon source in a similar manner as ylXYL+ (similar growth rate and lag phase) (Fig. 1a, b).

Both ylXYL+ and ylXYL+Obese were able to consume glucose and xylose when a mix of the two sugars was used as a carbon source. However, as previously shown with the heterologous genes [13], both strains present a partially sequential use of the two substrates. Moreover, the obese phenotype shows a higher biomass and a lower xylitol production than ylXYL+ (Fig. 1c for ylXYL+ and Fig. 1d for ylXYL+Obese).

### *Yarrowia lipolytica* ylXYL+ and ylXYL+Obese can produce lipids from xylose as a sole carbon source

**Fig. 2:**
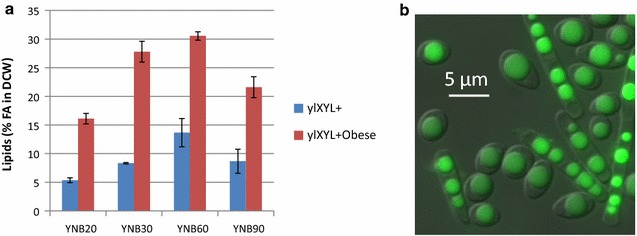
Lipid production by ylXYL+ and ylXYL+Obese strains in xylose. **a** Amount of lipids (% of fatty acids in the dry cell weight) produced in the ylXYL+ (blue) or ylXYL+Obese (red) strains grown in varying concentrations of xylose (YNB20, YNB30, YNB60, and YNB90; see “Methods”). **b** Fluorescence microscopy image showing the cells and their lipids stained with Bodipy in the YNB60 medium with the strain ylXYL+Obese

Nitrogen limitation is often required to boost lipid production in *Y. lipolytica*. Therefore, we tested the impact of different carbon/nitrogen ratios in the culture media (YNB20, YNB30, YNB60, and YNB90) on the growth and lipid production capacity of the modified *Y. lipolytica* strains using xylose as a sole carbon source (Fig. 2 and Table 1). After growing both strains in flasks, we found that YNB60 is the most convenient medium for lipid accumulation because a higher or lower ratio decreased lipid accumulation. On the one hand, ylXYL+Obese produces 2 times more lipids than ylXYL+. On the other hand, ylXYL+ produces more xylitol (75–100% more in YNB60 and YNB90) and citric acid (33–67% more in YNB60 and YNB90) than ylXYL+Obese (Additional file 1: Figure S2, Table S1). We can here conclude that ylXYL+Obese is the most convenient strain for the production of lipids, while ylXYL+ can be beneficial for the synthesis of biotechnological products, namely xylitol and citric acid.

**Table 1:** Titers, productivity, and yields in flasks with xylose as a sole carbon source. This table represents the titers in g/L, the productivity in g/Lh, and the yields in g/g of xylose after 6 days of flask culture with the engineered strains in YNB20, YNB30, YNB60, and YNB90 media. *X* biomass as DCW, *FA* fatty acids, *CA* citric acid, and *XOH* xylitol. The values represent the averages of two independent experiments. The values for the experiments that used ylXYL+ are on top, and those that used ylXYL+Obese are on the bottom

Strain	Media	S: xylose consumed (g/L)	X (g/L)	Y X/S (g/g)	FA (g/L)	Y FA/S (g/g)	FA P (g/Lh)	CA (g/L)	Y CA/S (g/g)	XOH (g/L)	Y XOH/S (g/g)
ylXYL+	YNB20	20 ± 0.00	9.53 ± 1.51	0.48	0.51 ± 0.04	0.03	0.004	0.00	0.00	0.18 ± 0.24	0.01
	YNB30	30 ± 0.00	9.23 ± 1.93	0.31	0.77 ± 0.17	0.03	0.005	1.64	0.05	2.13 ± 0.58	0.07
	YNB60	47 ± 5.04	16.7 ± 2.68	0.36	2.35 ± 0.58	0.05	0.016	4.24	0.09	2.62 ± 1.06	0.06
	YNB90	62 ± 0.81	18.26 ± 2.26	0.29	1.61 ± 0.58	0.03	0.011	2.71	0.04	2.77 ± 0.45	0.04
ylXYL+obese	YNB20	20 ± 0.00	10 ± 0.10	0.50	1.61 ± 0.09	0.08	0.011	0.04	0.00	0.00 ± 0.00	0.00
	YNB30	30 ± 0.00	13.53 ± 0.94	0.45	3.75 ± 0.02	0.13	0.026	0.00	0.00	0.00 ± 0.00	0.00
	YNB60	46 ± 0.28	19.06 ± 0.19	0.41	5.44 ± 0.09	0.12	0.037	2.55	0.06	1.49 ± 0.10	0.03
	YNB90	62 ± 1.09	22.83 ± 0.14	0.37	4.93 ± 0.45	0.08	0.034	2.04	0.03	1.32 ± 0.04	0.02

### ylXYL+ and ylXYK+Obese are able to grow and produce lipids in lignocellulosic hydrolysate

In this work, we obtained a lignocellulosic hydrolysate from agave bagasse, an abundant residue generated during the production of tequila (1 L of tequila generates approximately 10 kg of bagasse). Analysis of the composition of this hydrolysate showed that its carbohydrate content (87% of the DCW) is 15.5 ± 7.4 g/L glucose and 20.0 ± 6.9 g/L xylose and that it has acids (acetic acid 3.5 ± 1.5 g/L, lactic acid 3.7 ± 2.3, and formic acid 0.6 ± 0.3 g/L), glycerol (1.5 ± 0.7 g/L), and furfurals (3.5 ± 1.1 g/L).

We designed different culture media with different amounts of lignocellulosic hydrolysate (10, 20, 30, 40, 50, and 86%) while keeping the concentrations of the phosphate buffer and the nitrogen source constant (see “Methods”). Therefore, an increasing amount of hydrolysate represents a higher C/N ratio, but it also represents a higher amount of possible inhibitory compounds.

**Fig. 3:**
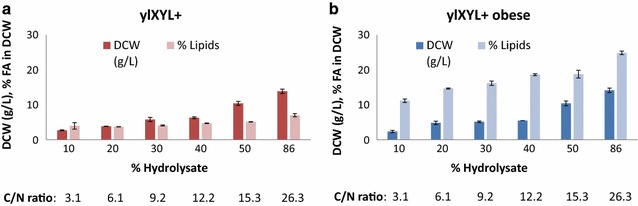
Lipid and biomass production by ylXYL+ and ylXYL+Obese strains in lignocellulosic hydrolysates. **a** Lipid (% FA in DCW) and biomass (g/L) production in the ylXYL+ strain when growing in media with different amounts of lignocellulosic hydrolysate (from 10 to 86%). **b** Lipid (% FA in DCW) and biomass (g/L) production in the ylXYL+Obese strain when growing in media with different amounts of lignocellulosic hydrolysate (from 10 to 86%). The values represent the average values and the standard deviation of at least two replicates. The C/N ratio for each condition is stated at the bottom of each bar

We found that ylXYL+ grew better with an increasing amount of hydrolysate, which seems to indicate a good tolerance to potentially toxic compounds (Fig. 3a). Generally, a higher C/N ratio resulted in a higher lipid content (which oscillated from 4 to 7%). Therefore, the total production of lipids was higher in the media with 86% hydrolysate.

ylXYL+Obese showed similar behavior to ylXYL+ (Fig. 3b); the produced biomass increased in parallel to the amount of hydrolysate as well as the lipid content. The lipid content increased from 11.5 to 24.5% of the DCW.

These results show that the modified *Y. lipolytica* strains are able to efficiently produce lipids directly from lignocellulosic hydrolysate.

### Metabolic engineering of xylose-consuming strains maximizes the conversion of sugars into lipids

**Fig. 4:**
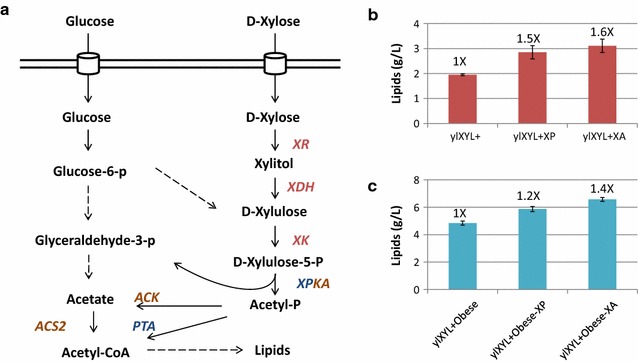
Xylose utilization pathway enhanced for lipid production. **a** Schematic representation of the pathway leading to the consumption of xylose. *XR* xylose reductase; *XDH* xylitol dehydrogenase; and *XK* xylulose kinase represent the xylose consumption pathway (in red). *XPKA* phosphoketolase, *ACK* acetate kinase, and *ACS2* acetyl-CoA synthase represent the XA pathway (in brown), while *XPKA* phosphoketolase and *PTA* phosphotransacetylase are the XP pathway (in blue). Dashed lines represent multiple metabolic steps. The bar graphs represent the lipid production (g/L) in the modified strains incorporating the XP or XA pathways compared to their parental strains, ylXYL+ (**b**) and ylXYL+Obese (**c**), when they were grown on xylose as a sole carbon source. The values represent the average values and the standard deviation of at least two replicates. The fold increase in relation with the parental strain is indicated on the top of each bar

Acetyl-CoA is the precursor molecule for fatty acid synthesis. It can be converted to malonyl-CoA and, together, they can be used by the FAS complex (*FAS1* and *FAS2*) to produce palmitic acid and stearic acid. In *Y. lipolytica*, the two major sources of acetyl-CoA are the enzyme *ACS2*, which uses acetate as substrate, and ATP citrate lyase (heterodimer of *ACL1* and *ACL2*), which uses citrate. However, other organisms have developed alternative pathways for acetyl-CoA production. Here, we have transferred two phosphoketolase pathways, named XP and XA here, which use xylulose-5-P, an intermediate in the xylose degradation pathway, as a substrate (Fig. 4a). The XP pathway is intended to convert xylulose-5-P to glyceraldehyde-3-P and acetyl-P via the *XPKA* enzyme (phosphoketolase) from *Aspergillus nidulans* and to convert the generated acetyl-P into acetyl-coA via *PTA* (phosphotransacetylase) from *Bacillus subtilis*. In the course of these experiments, the XP pathway increased fatty acid production from glucose in *Y. lipolytica* by 53% [23] and increased fatty acid production from xylose in the non-oleaginous fungus *A. gossypii* by 54% [24]. The XA pathway expresses the *XPKA* enzyme to produce acetyl-P and the *ACK* enzyme (acetate kinase) from *A. nidulans* to convert acetyl-P into acetate with the concomitant production of ATP. Thereafter, acetate can be converted into acetyl-CoA via the native *ACS2* enzyme.

We therefore generated strains ylXYL+XP, ylXYL+XA, ylXYL+Obese-XP, and ylXYL+Obese-XA and analyzed their capacity to produce lipids from xylose as a sole carbon source (media YNB60). The four strains showed a higher lipid production than their parental strains (Fig. 4b, c). Interestingly, the four strains were also able to produce a higher amount of biomass (ylXYL+XP 22%, ylXYL+XA 21%, ylXYL+Obese-XP 13%, and ylXYL+Obese-XA 18%) than their parental strains, and therefore, the lipid titers (grams of lipids per liter) were further increased. ylXYL+XP and ylXYL+XA produced 46 and 59% more lipids than ylXYL+ (Fig. 4b), respectively, while ylXYL+Obese-XP and ylXYL+Obese-XA produced 21 and 35% more lipids than ylXYL+Obese (Fig. 4c), respectively. We can here conclude that both strategies succeeded in the redirection of xylose towards acetyl-CoA and lipids, with slightly better results with the XA pathway. The higher efficiency of the XA pathway compared to the XP pathway had been reported for the production of fatty acid ethyl esters (FAEE) in *S. cerevisiae* from glucose [25]. We here selected ylXYL+Obese-XA as a good candidate to produce lipids from lignocellulosic hydrolysate in controlled conditions.

### Production of lipids in fed-batch fermentation from lignocellulosic hydrolysate in bioreactor-controlled conditions

**Fig. 5:**
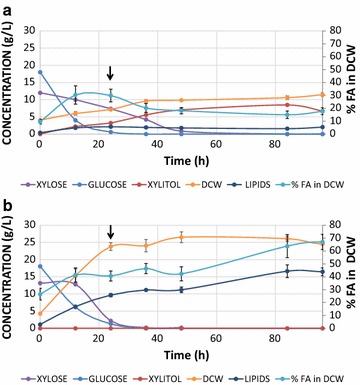
Lipid production in a bioreactor from lignocellulosic hydrolysate as a sole carbon source. Kinetics of the fed-batch bioreactor experiments for the wild type (**a**) and the engineered strain ylXYL+Obese-XA (**b**). Average concentrations with standard deviations are shown for the following compounds: lipids, DCW, xylitol, glucose, and xylose (g/L) and % of FA in the DCW. The arrows indicate the start of the fed (see “Methods”)

Based on the abovementioned experiments, ylXYL+Obese-XA, a strain overexpressing *XR*, *XDH*, and *XK* for the utilization of xylose, XPKA and ACK for the conversion of xylulose-5-P into acetate, and *DGA2* and *GPD1* for boosting TAG production and deletion of *POX1*-*6* and *TGL4* in order to avoid the degradation of fatty acids and TAGs, showed the best behavior for the production of lipids from xylose, one of the most abundant sugars in the lignocellulosic hydrolysate from agave. We therefore decided to test its ability to produce lipids from lignocellulosic hydrolysate in 2-L bioreactors, together with a control strain (the wild type). We performed fed-batch fermentations with an initial C/N ratio of 15 (30 g of sugars, 18% glucose, and 12% xylose exclusively from the hydrolysate) to promote biomass formation followed by the controlled addition, after 24 h, of 2 times concentrated hydrolysate at constant rate (60 g of sugars) in order to increase lipid production (overall C/N of 45). The fermentations run for 96 h, when all the sugars were consumed (45 g/L) and the production of biomass and lipids were stable. Both glucose and xylose were consumed in the fermentations (wild type, Fig. 5a and ylXYL+Obese-XA, Fig. 5b), being glucose quickly depleted from the broth in 24 h. As expected, xylose was degraded in both strains but much faster in ylXYL+Obese-XA (between 24 and 36 h) than in the wild type (between 48 and 84 h). Supplemented sugars in the feeding hydrolysate were consumed almost instantly, which promoted a longer steady state of biomass and lipid accumulation in both strains. The biomass production was much higher in ylXYL+Obese-XA (25.8 g/L, compared to 11.5 in the wild type), presumably due to the efficient incorporation of the xylose. This is also supported by the fact that the wild type accumulates 8.5 g/L of xylitol in the culture broth, originated by a non-complete degradation of xylose, while no xylitol is produced by the engineered strain. Similar behavior has been reported for the wild type growing in mixtures of glucose and xylose in flask [13]. Importantly, the lipid production reached 16.5 g/L in the engineered strain, 8.3 times more than the wild type (2 g/L). The maximum lipid content was 67% with a yield of 0.344 g lipids/g sugars and a maximum productivity of 0.185 g/L/h. Interestingly, this is the highest yield ever reported for the production of lipids in *Yarrowia lipolytica* and it is equivalent to the maximum theoretical yield (0.364 g lipids/g glucose). Such high yield can be explained by the presence of other components in the hydrolysate that can be also consumed to generate more lipids and/or biomass. 5.3 g/L of lactic acid, 2.4 g/L of acetic acid, and 1 g/L of glycerol present in the hydrolysate are degraded within 48, 12, and 12 h, respectively (data not shown). Importantly, the high lipid yield and productivity is achieved from a very low-cost lignocellulosic hydrolysate. In future approaches, further bioreactor optimization in high cell densities could potentially improve the lipid titer.

## Conclusions

In this work, we first showed that overexpression of the native *XDH*, *XR*, and *XK* genes in Po1d allowed it to grow in xylose as a sole carbon source. The same strategy was transferred to a strain that was modified to overproduce lipids and was able to efficiently convert xylose into oils. Importantly, this strain was still able to respond to the different C/N ratios, and it showed proper growth in low-nitrogen media, which was not the case for Po1f [20]. This feature allowed a boost in lipid production in culture media with a high C/N ratio. We thereafter tested whether these strains were able to use agave lignocellulosic hydrolysate, which is mainly composed of xylose and glucose, as a carbon source for growth and oil production, regardless of the presence of potentially toxic agents. Moreover, we engineered two additional pathways to generate acetyl-CoA, a precursor of lipids, from xylulose-5-P, an intermediate in the degradation of xylose. We found that both pathways increased lipid production from xylose, and we selected the best strain to grow in fed-batch bioreactor-controlled conditions using cheap lignocellulosic hydrolysate as a unique carbon source. Importantly, the fermentation conditions achieved very high lipid yield and productivity, even higher than those described in expensive and defined synthetic media.

Therefore, this work serves as a proof of concept for the improvement of the production of lipids by *Y. lipolytica* from lignocellulosic materials. In addition, it also creates room for further improvements via bioreactor condition optimization, where alternative fed-batch or continuous processes can be explored. Moreover, the designed strains can be tested in other lignocellulosic hydrolysates with different carbohydrate compositions and varying amounts of potentially toxic compounds.

This work allows us to reduce the costs associated with the production of lipid-derived compounds, such as biofuels or chemicals, as well as the cost of producing other compounds, such as organic acids, proteins (highly produced by *Y. lipolytica*), or novel compounds derived from acetyl-CoA (which is enhanced in the described strategy). The results presented in this paper bring us one step closer to a feasible fermentation-based economy.

## Methods

### Strains, cultures, and media

The *Y. lipolytica* strains used in this study were Po1d (Ura^−^ Leu^−^) [whose parental strain is the wild-type strain W29 (ATCC20460)] and its derived strains. The prototrophic strains generated in this study are listed in Additional file 1: Table S1. Media and growth conditions for *Escherichia coli* and *Y. lipolytica* have been described elsewhere [26]. Minimal medium contained 0.17% (wt/vol) yeast nitrogen base without amino acids and ammonium sulfate (YNBww, DIFCO); 2, 3, 6, or 9% xylose (wt/vol; Merck, Fontenay-sous-Bois Cedex, France) for YNB20, YNB30, YNB60, and YNB90, respectively; 0.15% (wt/vol) NH_4_Cl; and 50 mM phosphate buffer (pH 6.8). Media with glucose were prepared in a similar way as YNB by substituting 2% xylose with 2% glucose. Media containing both sugars included 3% glucose and 3% xylose. The media with hydrolysate contained 10, 20, 30, 40, 50, and 86% (v/v) of hydrolysate, 0.17% (wt/vol) yeast nitrogen base (YNBww), 0.15% (wt/vol) NH_4_Cl, and 50 mM phosphate buffer (pH 6.8).

To evaluate lipid production from bagasse hydrolysates, batch bioreactor cultures were performed in duplicate in 3-L Applikon glass bioreactors using My Control consoles at 1 vvm aeration, 750 rpm agitation, and pH 6.8. Working volume was 2 L. Fed-batch cultures were performed following this strategy, beginning with 1 L of medium in a batch mode at the same conditions but using a concentration of 0.3% (wt/vol) NH_4_Cl in order to start at a C/N ratio of 15. After 24 h 1 L of fed medium containing 60 g/L of total sugars from lignocellulosic hydrolysate, 0.17% (w/v) YNBww and 50 mM phosphate buffer (pH 6.8) were added at a constant flow of 0.35 mL/min. The hydrolysate was concentrated two times for the fed by evaporation. We did not concentration further the hydrolysate because it showed solid precipitations and thus associated changes in soluble composition.

### Lignocellulosic hydrolysate

Agave bagasse was obtained from a tequila factory in Jalisco, Mexico. It was dried to a constant weight at 70 °C to measure its humidity prior to hydrolysis and characterization. Bagasse hydrolysis was performed through a chemical and enzymatic procedure according to patent application MX/E/2015/084126. Finally, a liquid hydrolysate was obtained. Furfurals, acids, alcohols, and sugar content in the liquid hydrolysate were measured by HPLC.

### Cloning and expression of heterologous and endogenous genes

The endogenous genes *XDH* (YALI0E12463), *XR* (YALI0D07634), and *XK* (YALIF10923) were amplified from Po1d genomic DNA using the appropriate primers (Additional file 1: Table S2). The PCR fragments were digested using *Bam*HI/*Avr*II and were inserted into the plasmid JMP62 TEF [27] at the corresponding sites with an LEU2, URA3, or HYG marker. The heterologous genes XPKA (AN4913.2) and ACK (AN4914.2) from *A. nidulans* and PTA (AIY95081.1) from *B. subtilis* were codon optimized, synthesized by GenScript, and cloned into JMP62 TEF-derived plasmids with a URA or LEU marker. The complete list of genes used in this study is shown in Additional file 1: Table S3.

Expression vectors were digested with NotI, purified on a gel, and used for transformation as described in previous works [28]. Depending on their genotype, transformants were selected on YNBG (1% glucose) media, supplemented with uracil or leucine (for auxotrophic strains), or on YPDHyg. Positive transformants were checked by PCR. The removal of the selection marker was carried out via the LoxP-Cre system, which is widely used in *Yarrowia* [29]. The Po1d (JMY195) strain transformed with *ylXR*, *ylXDH*, and *ylXK* was named ylXYL+, while the obese strain JMY3501 [14], which is highly modified for enhancing lipid accumulation, was further transformed with the same xylose pathway genes and was named ylXYL+Obese.

Restriction enzymes were obtained from OZYME (Saint-Quentin-en-Yvelines, France). PCR amplifications were performed in an Eppendorf 2720 thermal cycler with GoTaq DNA polymerases (Promega). PCR fragments were purified with a Qiagen Purification Kit (Qiagen, Hilden, Germany). All reactions were performed according to the manufacturers’ instructions.

### Determination of DCW, growth rate, sugars, acids, and alcohols

To determine DCW in the flask experiments, 2 mL of culture was washed and lyophilized in a pre-weighed tube. The differences in weight corresponded to the mg of cells found in 2 mL of culture.

Growth tests were performed in 100 µL cultures in 96-well plates, with constant shaking, in the presence of 0.5% glucose or xylose as a carbon source. Growth was monitored by measuring the optical density (OD_600 nm_) at different intervals with a microtiter plate reader (Biotek, Colmar, France). For each strain and set of conditions, we used at least two biological replicates. A 2 mL preculture was grown for 16 h and was used to inoculate the cultures at an OD600 of 0.2. The growth rate was calculated in the exponential phase for each strain and condition. The lag phase was determined as the time at which the exponential phase began.

Glucose, xylose, xylitol, and citric acid were identified and quantified by HPLC (UltiMate 3000, Dionex-Thermo Fisher Scientific, UK) using an Aminex HPX87H column coupled to UV (210 nm) and RI detectors. The column was eluted with 0.01 N H_2_SO_4_ at room temperature and a flow rate of 0.6 mL/min. Identification and quantification were achieved via comparisons to standards. Before being subjected to HPLC analysis, samples were filtered on membranes of 0.45-μm pore size. Citric acid and isocitric acid are undistinguishable in our methods and thus when citric acid is referred in the text the value represents the sum of all citric acid forms.

### Lipid quantification

Lipids from aliquots of 10–20 mg of cells were converted into their methyl esters with freeze-dried cells according to Browse et al. [30] and were used for gas chromatography (GC) analysis. GC analysis of fatty acid (FA) methyl esters was performed with a Varian 3900 instrument equipped with a flame ionization detector and a Varian FactorFour vf-23 ms column, where the bleed specification at 260 °C is 3 pA (30 m, 0.25 mm, 0.25 μm). FAs were identified by comparison to commercial FA methyl ester standards (FAME32; Supelco) and quantified by the internal standard method, which involves the addition of 50 μg of commercial C17:0 (Sigma).

### Microscopic analysis

The image was acquired using a Zeiss Axio Imager M2 microscope (Zeiss, Le Pecq, France) with a 100× objective and Zeiss filters 45 and 46 for fluorescence microscopy. Axiovision 4.8 software (Zeiss, Le Pecq, France) was used for image acquisition. Lipid body visualization was performed after adding Bodipy^®^ Lipid Probe (2.5 mg/mL in ethanol; Invitrogen) to the cell suspension (*A*_600_ of 5) with incubation for 10 min at room temperature.

## Additional file

**Additional file 1: Figure S1.** Growth curves of Po1d (in glucose and xylose media), ylXYL+ and ssXYL+ (in xylose media) when grown on microplate (0.5% of carbon source). **Figure S2.** Production of xylitol and citric acid by the modified strains grown on xylose as a sole carbon source. Values of the production of xylitol (red) and citric acid (green) when the strains ylXYL+ (A) or ylXYL+Obese (B) were grown in media with different concentrations of xylose (YNB20, YNB30, YNB60, and YNB90; see “Methods”). The values represent the average values and the standard deviation of at least two replicates. **Table S1.** List of prototrophic strains used in this study. **Table S2.** List of primers used in this study. **Table S3.** List of genes modified in this study.

## Abbreviations

TAG: triacylglycerol
FAEE: fatty acid methyl ester
DCW: dry cell weight
XR: xylose reductase
XDH: xylitol dehydrogenase
XK: xylulose kinase
XPKA: phosphoketolase
ACK: acetate kinase
ACS2: acetyl-CoA synthase
C/N: ration carbon to nitrogen
C5: pentose
