# Supplementary material for: Engineering Yarrowia lipolytica to enhance lipid production from lignocellulosic materials
Source: Biotechnol Biofuels. 2018 Jan 22;11:11. doi: 10.1186/s13068-018-1010-6 (PMC5776775; doi:10.1186/s13068-018-1010-6)
Supplement: Supplementary file 1 — Additional file 1: Figure S1. Growth curves of Po1d (in glucose and xylose media), ylXYL+ and ssXYL+ (in xylose media) when grown on microplate (0.5% of carbon source). Figure S2. Production of xylitol and citric acid by the modified strains grown on xylose as a sole carbon source. Values of the production of xylitol (red) and citric acid (green) when the strains ylXYL+ (A) or ylXYL+Obese (B) were grown in media with different concentrations of xylose (YNB20, YNB30, YNB60, and YNB90; see “Methods”). The values represent the average values and the standard deviation of at least two replicates. Table S1. List of prototrophic strains used in this study. Table S2. List of primers used in this study. Table S3. List of genes modified in this study. [file 13068_2018_1010_MOESM1_ESM.pptx]

## Slide 1
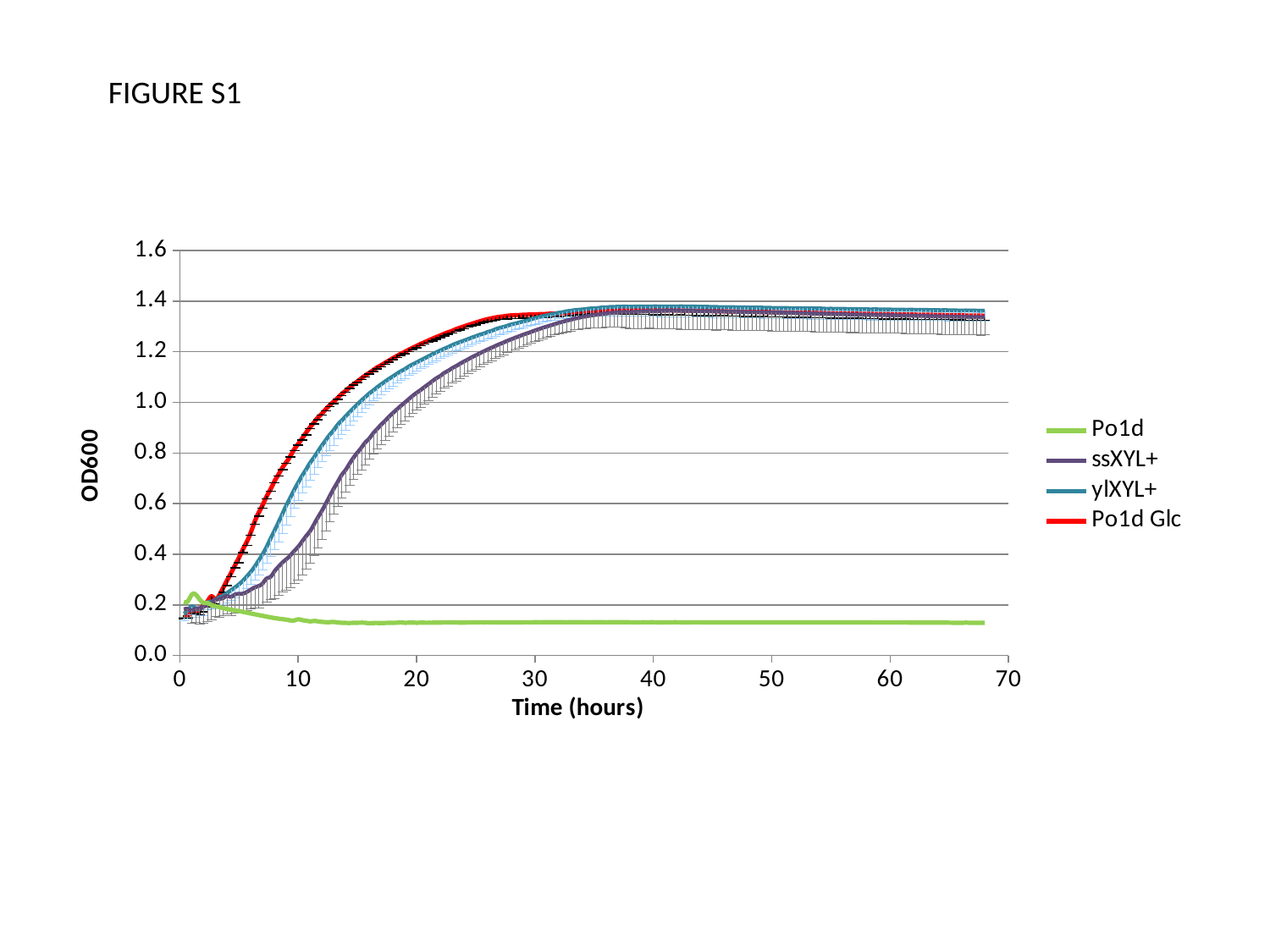

FIGURE S1
### Chart
| Category | Po1d | ssXYL+ | ylXYL+ | Po1d Glc |
|---|---|---|---|---|

## Slide 2
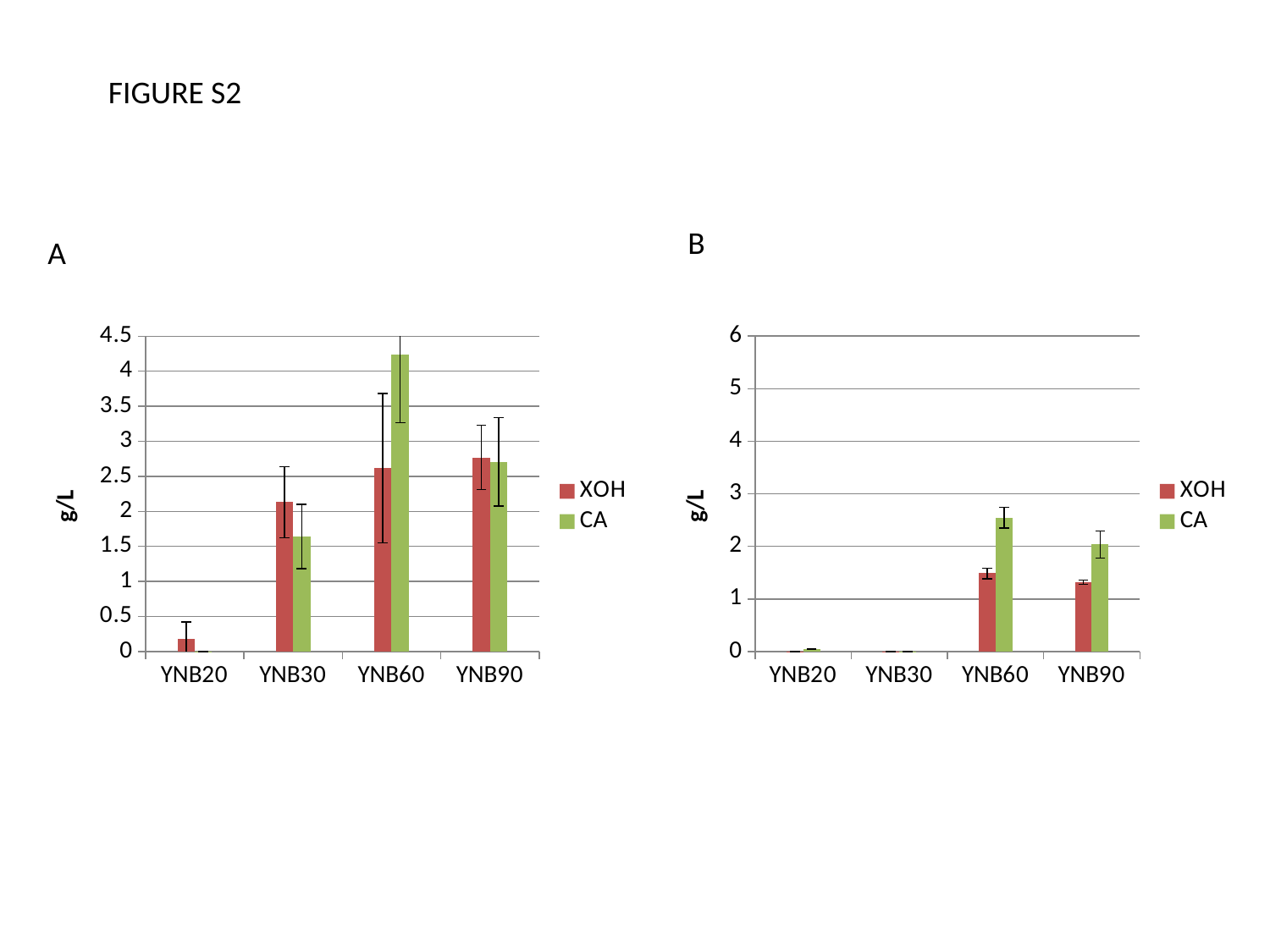

FIGURE S2
B
A
### Chart
| Category | XOH | CA |
|---|---|---|
| YNB20 | 0.1755709334753592 | 0.00010000000000000011 |
| YNB30 | 2.131352065915179 | 1.6421203670985651 |
| YNB60 | 2.618038254666838 | 4.2359895701760255 |
| YNB90 | 2.769533708147189 | 2.7083249001871392 |
### Chart
| Category | XOH | CA |
|---|---|---|
| YNB20 | 0.00010000000000000011 | 0.04331187676680486 |
| YNB30 | 0.00010000000000000011 | 0.00010000000000000011 |
| YNB60 | 1.48807707872597 | 2.5493573321232974 |
| YNB90 | 1.3207349899250165 | 2.036742624829126 |

## Slide 3
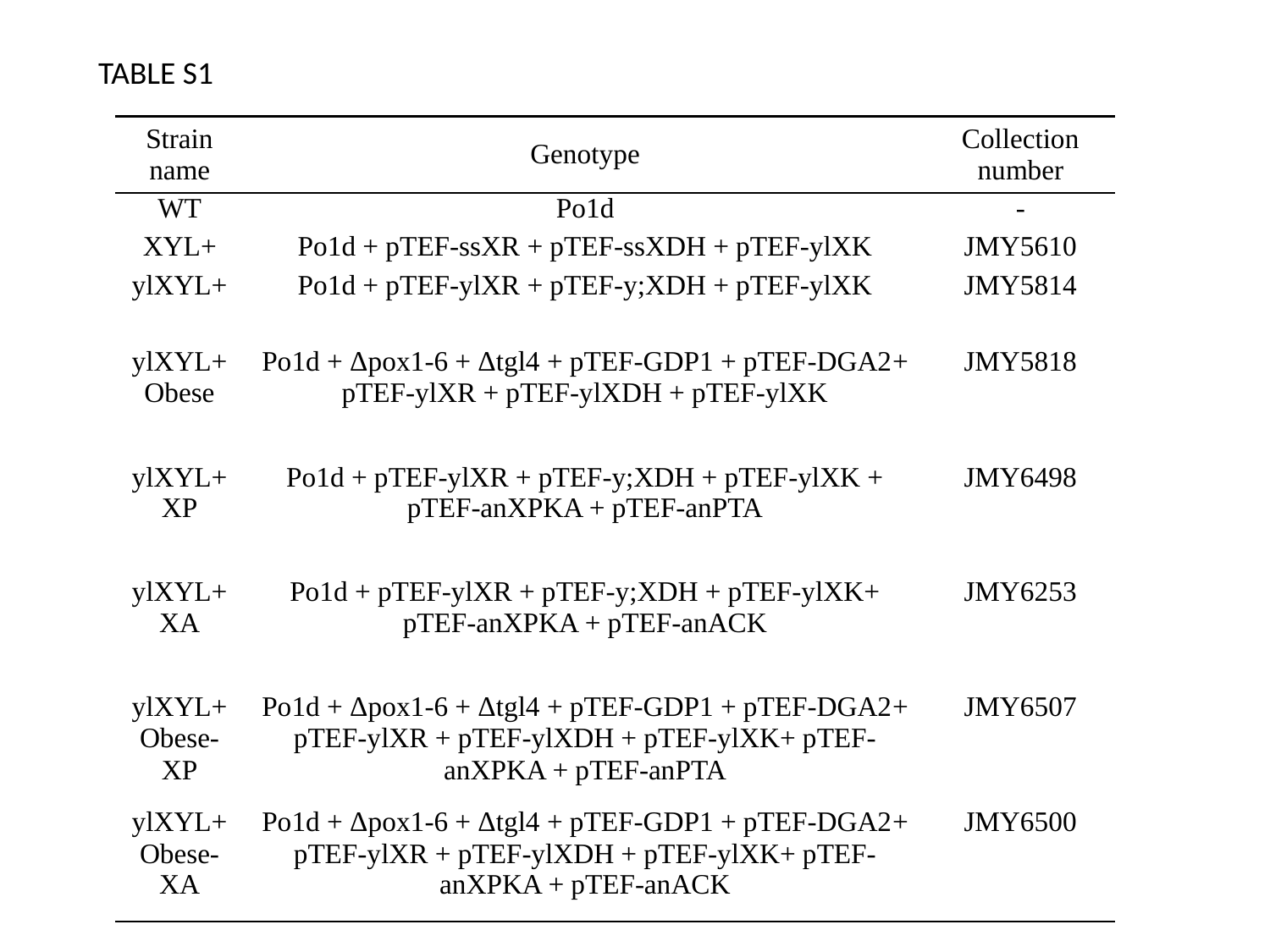

TABLE S1
| Strain name | Genotype | Collection number |
| --- | --- | --- |
| WT | Po1d | - |
| XYL+ | Po1d + pTEF-ssXR + pTEF-ssXDH + pTEF-ylXK | JMY5610 |
| ylXYL+ | Po1d + pTEF-ylXR + pTEF-y;XDH + pTEF-ylXK | JMY5814 |
| ylXYL+ Obese | Po1d + Δpox1-6 + Δtgl4 + pTEF-GDP1 + pTEF-DGA2+ pTEF-ylXR + pTEF-ylXDH + pTEF-ylXK | JMY5818 |
| ylXYL+ XP | Po1d + pTEF-ylXR + pTEF-y;XDH + pTEF-ylXK + pTEF-anXPKA + pTEF-anPTA | JMY6498 |
| ylXYL+ XA | Po1d + pTEF-ylXR + pTEF-y;XDH + pTEF-ylXK+ pTEF-anXPKA + pTEF-anACK | JMY6253 |
| ylXYL+ Obese- XP | Po1d + Δpox1-6 + Δtgl4 + pTEF-GDP1 + pTEF-DGA2+ pTEF-ylXR + pTEF-ylXDH + pTEF-ylXK+ pTEF-anXPKA + pTEF-anPTA | JMY6507 |
| ylXYL+ Obese- XA | Po1d + Δpox1-6 + Δtgl4 + pTEF-GDP1 + pTEF-DGA2+ pTEF-ylXR + pTEF-ylXDH + pTEF-ylXK+ pTEF-anXPKA + pTEF-anACK | JMY6500 |

## Slide 4
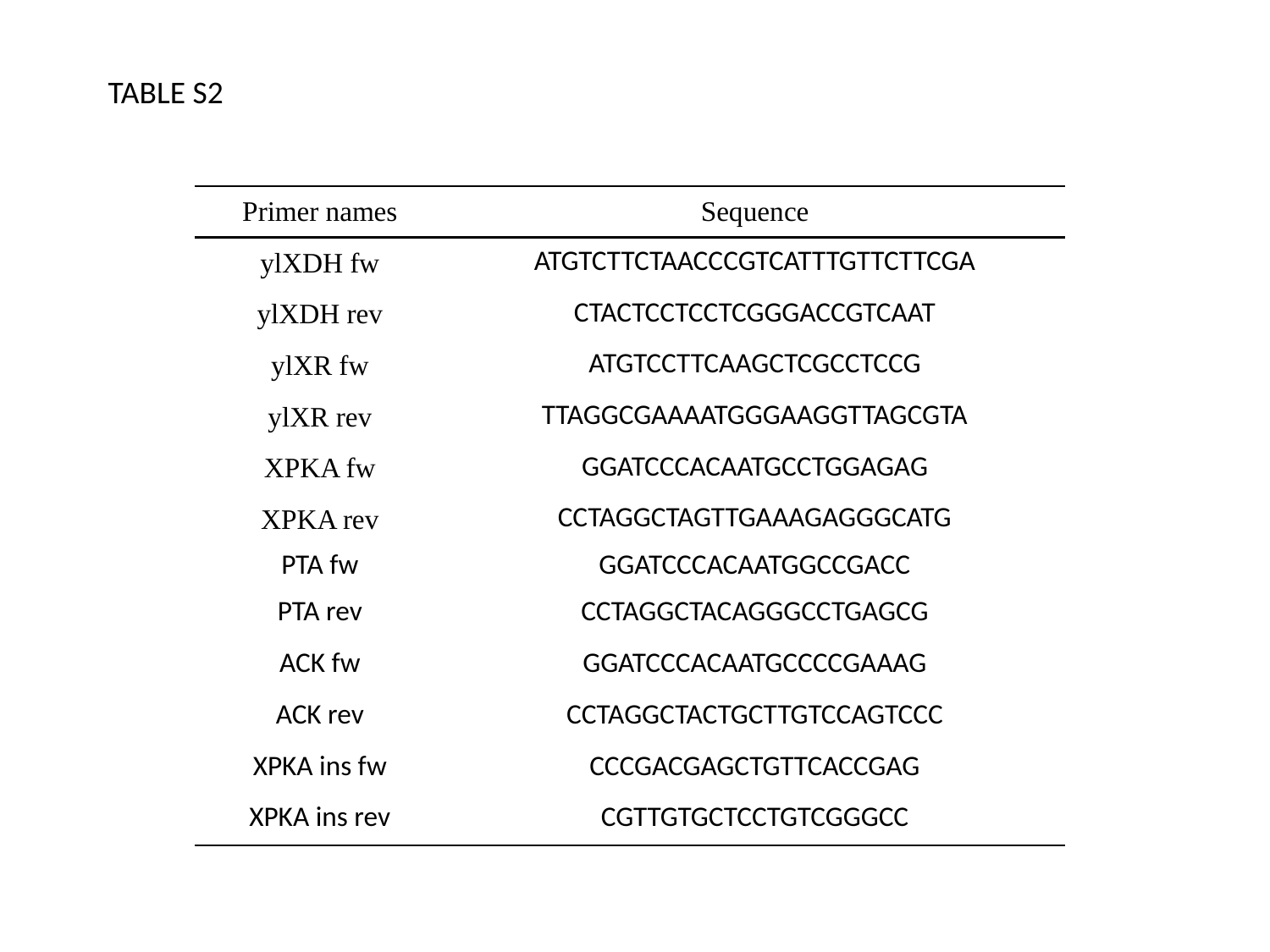

TABLE S2
| Primer names | Sequence |
| --- | --- |
| ylXDH fw | ATGTCTTCTAACCCGTCATTTGTTCTTCGA |
| ylXDH rev | CTACTCCTCCTCGGGACCGTCAAT |
| ylXR fw | ATGTCCTTCAAGCTCGCCTCCG |
| ylXR rev | TTAGGCGAAAATGGGAAGGTTAGCGTA |
| XPKA fw | GGATCCCACAATGCCTGGAGAG |
| XPKA rev | CCTAGGCTAGTTGAAAGAGGGCATG |
| PTA fw | GGATCCCACAATGGCCGACC |
| PTA rev | CCTAGGCTACAGGGCCTGAGCG |
| ACK fw | GGATCCCACAATGCCCCGAAAG |
| ACK rev | CCTAGGCTACTGCTTGTCCAGTCCC |
| XPKA ins fw | CCCGACGAGCTGTTCACCGAG |
| XPKA ins rev | CGTTGTGCTCCTGTCGGGCC |

## Slide 5
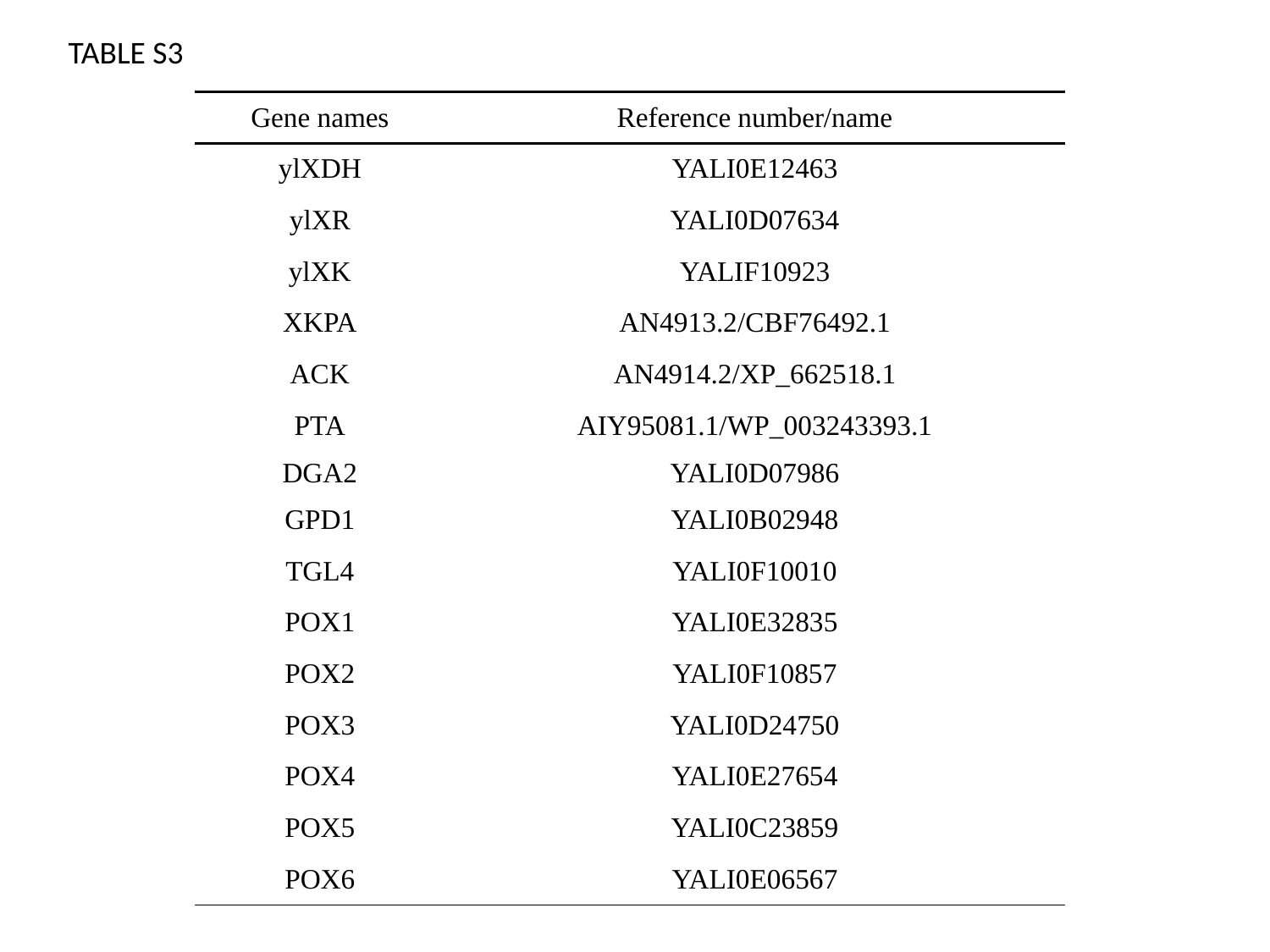

TABLE S3
| Gene names | Reference number/name |
| --- | --- |
| ylXDH | YALI0E12463 |
| ylXR | YALI0D07634 |
| ylXK | YALIF10923 |
| XKPA | AN4913.2/CBF76492.1 |
| ACK | AN4914.2/XP\_662518.1 |
| PTA | AIY95081.1/WP\_003243393.1 |
| DGA2 | YALI0D07986 |
| GPD1 | YALI0B02948 |
| TGL4 | YALI0F10010 |
| POX1 | YALI0E32835 |
| POX2 | YALI0F10857 |
| POX3 | YALI0D24750 |
| POX4 | YALI0E27654 |
| POX5 | YALI0C23859 |
| POX6 | YALI0E06567 |
